# The Origins of Mental Toughness – Prosocial Behavior and Low Internalizing and Externalizing Problems at Age 5 Predict Higher Mental Toughness Scores at Age 14

**DOI:** 10.3389/fpsyg.2016.01221

**Published:** 2016-08-24

**Authors:** Dena Sadeghi Bahmani, Martin Hatzinger, Markus Gerber, Sakari Lemola, Peter J. Clough, Sonja Perren, Kay von Klitzing, Agnes von Wyl, Edith Holsboer-Trachsler, Serge Brand

**Affiliations:** ^1^Center for Affective, Stress and Sleep Disorders, Psychiatric Clinics of the University of BaselBasel, Switzerland; ^2^Department of Adult Psychiatry, Psychiatric Services SolothurnSolothurn, Switzerland; ^3^Department of Sport, Exercise and Health, Division of Sport and Psychosocial Health, University of BaselBasel, Switzerland; ^4^Department of Psychology, University of WarwickCoventry, UK; ^5^Department of Psychology, Manchester Metropolitan UniversityManchester, UK; ^6^Department of Teacher Education, University of KonstanzKonstanz, Germany; ^7^Department of Child and Adolescent Psychiatry, Psychotherapy, and Psychosomatics, University of LeipzigLeipzig, Germany; ^8^Institute of Psychology, University of ZurichZurich, Switzerland

**Keywords:** mental toughness, sleep, origins, long-term, pro-social behavior, internalizing problems, externalizing problems

## Abstract

**Background:** The concept of mental toughness (MT) has gained increasing importance among groups other than elite athletes by virtue of its psychological importance and explanatory power for a broad range of health-related behaviors. However, no study has focused so far on the psychological origins of MT. Therefore, the aims of the present study were: to explore, to what extent the psychological profiles of preschoolers aged five were associated with both (1) MT scores and (2) sleep disturbances at age 14, and 3) to explore possible gender differences.

**Method:** Nine years after their first assessment at age five (preschoolers), a total of 77 adolescents (mean age: 14.35 years; *SD* = 1.22; 42% females) took part in this follow-up study. At baseline, both parents and teachers completed the Strengths and Difficulties Questionnaire (SDQ), covering internalizing and externalizing problems, hyperactivity, negative peer relationships, and prosocial behavior. At follow-up, participants completed a booklet of questionnaires covering socio-demographic data, MT, and sleep disturbances.

**Results:** Higher prosocial behavior, lower negative peer relationships, and lower internalizing and externalizing problems at age five, as rated by parents and teachers, were associated with self-reported higher MT and lower sleep disturbances at age 14. At age 14, and relative to males, females had lower MT scores and reported more sleep disturbances.

**Conclusion:** The pattern of results suggests that MT traits during adolescence may have their origins in the pre-school years.

## Introduction

Clough et al. (2002) formulate the concept of Mental toughness (MT) in its present form and since then, the concept of MT has gained increasing interest by virtue of its psychological importance and explanatory power with respect to psychological concepts such as coping with stress, self-esteem, and motivation, and with respect to a broad range of health-related behaviors (**Table 1**) (Dewhurst et al., 2012; Gerber et al., 2013b; Perry et al., 2013; Crust et al., 2014; Stamp et al., 2015). MT refers to an individual’s capacity to be consistently successful in coping with difficult life circumstances and comprises the following dimensions: Control (own life and emotions), Commitment (to personal aims and achievements), Challenge (considering changes in life not as threats but as challenges), and Confidence (in own abilities and in other people) (Clough et al., 2002; Perry et al., 2013). Thus, MT refers to the tendency to appraise threats and pressure as opportunities to thrive (Thelwell et al., 2005), actively to seek and approach challenges (Crust, 2008), and successfully to overcome setbacks and difficulties (Clough et al., 2002; Dewhurst et al., 2012). Thus, as shown in and apparent from **Table 1**, MT embodies a range of cognitive-emotional processes closely involved in coping with stress, motivation, self-esteem, unexpected events, and social settings (confidence in other people).

**Table 1:** Overview of Mental toughness (MT) traits in relation to other psychological concepts (the list doesn’t claim to be complete).

		Mental toughness			
		
Concept	Authors	Control	Commitment	Confidence	Challenge
Resilience	Skala and Bruckner, 2014			X	X
Hardiness	Kobasa et al., 1982	X		X	X
Cognitive appraisal of stress	Lazarus and Folkman, 1984	X			X
Perceived Stress Scale	Cohen et al., 1983	X		X	X
Self-esteem	Rosenberg, 1965			X	
Self-efficacy	Bandura, 1977	X		X	
Self-regulation	Sameroff and Rosenblum, 2006; Keefer et al., 2013	X		X	X
Intrinsic motivation	Ryan and Deci, 2000	X	X	X	
Expectancy-value theory	Wigfield and Eccles, 2000		X	X	


Initially, studies in this field focused on MT in elite athletes (Loehr, 1994; Fourie and Potgieter, 2001; Jones et al., 2002, 2007; Thelwell et al., 2005; Crust, 2007, 2008; Clough and Strycharczyk, 2012); these studies showed that mentally tough athletes were able to cope with stress during a competition and to remain more focused and confident (Levy et al., 2006; Crust, 2007; Mack and Ragan, 2008; Nicholls et al., 2008; Kaiseler et al., 2009; Sheard, 2009; Crust and Azadi, 2010). However, more recent studies have applied the concept of MT to other groups such as healthy older adolescents (Gerber, 2011; Gerber et al., 2012, 2013a, 2015a,b; Brand et al., 2014a,b), healthy younger adolescents (Brand et al., 2016a,b), university students (Stamp et al., 2015), lower, middle, and senior managers, and clerical/administrative workers in early, middle and late adulthood (Marchant et al., 2009; Perry et al., 2013), as well as those working in education (Crust et al., 2014), and the military (Arthur et al., 2015). All these studies have shown higher MT scores to be associated with better coping with stress (Gerber et al., 2013a,b), with better sleep quality assessed both subjectively (Brand et al., 2014b) and objectively (Brand et al., 2014a), and with better physical performance (Crust and Clough, 2005; Gerber et al., 2012). Further, we showed that patients with multiple sclerosis (MS) at illness onset (mean age = 32.3 years) reported similar MT traits as healthy adolescents and young adults did (Sadeghi Bahmani et al., 2016).

To date, however, no study has considered the origins of MT by asking what psychological dimensions in childhood might predict MT traits in adolescence. The aim of the present study was therefore to address this question. To this end participants from a previous study when they were 5 years old and at kindergarten (Hatzinger et al., 2007, 2008, 2010; Brand et al., 2015) were contacted at age 14 and assessed once again. We believe that the present study has the potential to shed new light on the origins and development of MT, a psychological attribute, which appears to underlie a broad range of positive behaviors. We further hold that with the present study attention in developmental psychology might shift from psychopathology toward salutogenic dimensions.

In this regard, a literature search on the search engine Pubmed with the items ‘resilience’, ‘hardiness’, or ‘mental toughness’ in combination with ‘development’ produced very few results (note that the concepts of MT, resilience, and hardiness seem to share a common basis but without being synonyms; see **Table 1**). For ‘hardiness’, no study could be identified in combination with the term ‘development’. With regard to ‘resilience’ and ‘development’, the following points were identified: resilience is understood as an individual’s skill in successfully adapting to stress and adversity (Skala and Bruckner, 2014). Interpersonal factors associated with resilience include male gender, higher intelligence, aspects of character, temperament, and genes. Family factors include stable and positive relations with an adult, while a broader social environmental factor is being integrated into a community. Concerning the development of resilience, (Masten et al., 1999, 2004; Masten, 2004; Masten and Cicchetti, 2012; Masten and Tellegen, 2012) identified higher IQ and favorable parenting as factors with the potential to protect child development in the context of severe adversity. They also emphasized the relevance of adaptive resources, planfulness/future motivation, autonomy, adult support, and coping skills as possible factors underlying resilience and successful development. Additionally, Masten and Tellegen (2012) found that resilient adults reported high quality relationships with parents and other adults and good cognitive and socio-emotional skills during their childhood, while Sameroff and Rosenblum (2006) identified poor parenting, antisocial peers, low-resource communities, and economic hardship as the main factors impairing resilience.

Thus, while research examining the impact of hardiness and resilience on child development is scarce, and while the concept of MT offers a basis for integrating a broad range of coping literature into a common framework (**Table 1**), it must be noted that most previous studies have focused on the development of psychopathology. In fact, there is some evidence that psychopathology in preschoolers may persist over time and that childhood psychopathology could predict psychological difficulties in adolescence (Giedd et al., 1999; Paus et al., 1999, 2008; Caye et al., 2016). In particular, attention has been given to whether behavioral problems such as internalizing and externalizing behavior in preschoolers could predict psychiatric problems in adolescence and adulthood. However, externalizing problems have been more frequently investigated than internalizing problems and, in general, results indicated greater stability over time for externalizing than for internalizing behavior problems (Pihlakoski et al., 2006). Pihlakoski et al. (2006) have also shown that externalizing problems in boys and girls at age three strongly predicted both externalizing and internalizing problems at 12 years. Externalizing disorders are characterized by disruptive, disobedient, and harmful behaviors that are often manifested physically (e.g., in aggressive, impulsive, and non-compliant behavior; Weisz and Weiss, 1991), and seemed to exhibit considerable stability over time throughout development (Pihlakoski et al., 2006). Furthermore, externalizing behaviors have been associated with social aggression, disruptive behavior, a perceived lack of constraint, and risky behaviors; in particular, aggressive and destructive behaviors in early childhood predicted later problems (Pihlakoski et al., 2006). In addition, evidence indicated that childhood psychopathology was associated with higher rates of early substance use and problem substance use (King et al., 2004). Externalizing disorders (e.g., conduct problems and ADHD) have been found to have the strongest impact on later tobacco use, and children displaying aggressive behavior at 5 years were more likely to consume tobacco 14 years later (though, surprisingly, no association was found between externalizing problems and a prediction of DSM-IV nicotine dependence at 21-year follow-up; Fischer et al., 2012). However, in general, children with an early onset of conduct problems (onset in preschool) and a high degree of continuity seemed to have a much more negative prognosis than children with a late onset (adolescence) (McMahon, 1999). In conclusion, psychopathology such as externalizing problems in childhood and adolescence appeared to predict unfavorable behaviors such as tobacco consumption in later life (Fischer et al., 2012).

Internalizing disorders are characterized by feelings of sorrow, guilt, worry, and somatization (Weisz and Weiss, 1991), and children with internalizing disorders display reactions such as social withdrawal, a lack of pleasure in enjoyable activities, and a lack of energy (Cicchetti and Toth, 1998). Internalizing problems were linked to social deficits (e.g., submissive and inhibited interaction), poor interaction with the peer-group, social isolation and development of a negative self-concept (Fischer et al., 2012) and might lead to internalizing disorders such as depression and anxiety (McMahon, 1999). About 2–3% of children and 6–8% of adolescents suffer from depression and the lifetime prevalence of depression during adolescence was in the range 15–20% (McMahon, 1999). The estimated prevalence of anxiety disorders in childhood and adolescence varied from 9 to 21%. Girls were twice as likely to experience an anxiety disorder (McMahon, 1999), a trend recently further confirmed, in that internalizing problems had increased among recent cohorts of girls as compared to previous cohorts, but not among boys (Bor et al., 2014). Yet, findings with respect to internalizing behaviors are less consistent than those for externalizing behaviors; this may be due to young children’s limited ability to express anxiety and depression. Moreover, parents seemed to have difficulties in recognizing these emotions in their preschool children (Pihlakoski et al., 2006). Nevertheless, internalizing problems in 2–5 years young children have proved to be relatively stable over a 2-year follow-up period (Pihlakoski et al., 2006).

In addition, there is evidence that peer victimization (e.g., experiencing frequent verbal or physical bullying by peers) in middle childhood was a relevant predictor of internalizing behavior problems and psychological disorders during adolescence (Schwartz et al., 2015). There have been several indications of a moderate link between peer victimization and some form of internalizing behavior problem such as symptoms of depression, anxiety, loneliness, and withdrawal (Schwartz et al., 2015). More specifically, Schwartz et al. (2015) provided evidence that peer victimization in middle childhood could act as a key marker of disorders at later stages of development. These authors found that children who experienced frequent peer victimization in middle childhood were significantly more likely to meet criteria for a major depressive disorder during late adolescence.

Additionally, researchers have examined the long-term influence of sleep disturbances on several psychological problems. Existing research has shown that sleep problems were persistent and that individual differences in sleep problems were highly stable over time (Wong, 2010; Wong et al., 2010). Thus, Wong (2010) and Wong et al. (2010) found that having trouble sleeping at age 3 to 8 years was significantly associated with self-reported sleep problems at age 11 to 17. Indeed those who had trouble sleeping in childhood, compared to those having no trouble sleeping, had an almost 2.5-fold greater likelihood of having trouble sleeping in adolescence (Brand et al., 2015 for extensive overview).

In summary, previous research has focused on the predictive value of internalizing and externalizing problems, and sleep disturbances during childhood for psychopathology traits in adolescence. However, no evidence is available as regards the predictive value of internalizing and externalizing problems during childhood for psychological constructs such as MT in adolescence. Therefore, the aim of the present longitudinal study was to shed some light into this issue. We hold that the present data have the potential to add to the existing literature in important ways: first, compared to other psychological constructs as listed in **Table 1**, the concept of MT has been established only 1.5 decades ago, and to the best of our knowledge, no research has focused on psychological constructs as precursors of adolescent MT. Second, in our opinion, the concept of MT deserves further research, as it has the potential to cover a broad range of cognitive-emotional concepts such as coping, self-esteem, motivation, and social confidence in one single construct (see **Table 1**).

Given the lack of previous research, we drew upon findings relating to psychopathology in formulating our hypotheses. Thus, following others (Wong et al., 2010; Fischer et al., 2012; Settles et al., 2012), we anticipated that positive psychological traits evident in childhood (low externalizing and internalizing problems, high prosocial behavior) would predict higher MT scores and also lower sleep disturbances at age 14. Furthermore, based on previous research (Brand et al., 2014a,b), we expected that higher MT scores would be associated with lower sleep disturbances at age 14. Finally, we expected that compared to boys, girls would report lower MT scores (Brand et al., 2016a,b) and more sleep disturbances (Armitage and Hoffmann, 2001; Brand et al., 2016b; Mong and Cusmano, 2016).

## Materials and Methods

### Procedure

As described elsewhere (Brand et al., 2015), children participating in this study were assessed during their 1st year in kindergarten (when they were 5 years old; Hatzinger et al., 2007, 2008, 2010). These children were contacted again at age 14. Participants completed self-rating questionnaires covering socio-demographic information, MT and sleep (see below). The general purpose of the follow-up study was explained to the adolescents and their parents. Prior to entry to the study both adolescents and their parents were asked to sign an informed consent form. The study protocol was carried out in accordance with the Declaration of Helsinki and was approved by the local ethics committee.

Parts of the ongoing longitudinal study have been already published. Specifically, Brand et al. (2015) showed that sleep quality at the age of five predicted psychological traits in areas such as peer relationships and success at coping with stress while, surprisingly, sleep at the age of 14 years was unrelated. In the present study, we focused on the associations between participants’ psychological traits at age five (SDQ, parents’ and teachers’ ratings; see below) and participants’ self-rated MT and sleep disturbances at age 14. This pattern of associations has not been examined so far. Thus, the present data are novel.

### Sample

The core sample has been described in detail elsewhere (Perren et al., 2006; Brand et al., 2015). Briefly, preschoolers of kindergartens of Basel (Basel, Switzerland) were assessed as regards their subjective and objective sleep parameters, the level of saliva cortisol under baseline [cortisol awakening response (CAR)] and under challenge conditions (a modified social stress test), and their psychological functioning [Strengths and Difficulties Questionnaire (SDQ, see below for more details; (Goodman, 1997): internalizing and externalizing problems, hyperactivity, negative peer relationship, prosocial behavior], as assessed via parents’ and teachers’ ratings. Of the 95 children at age five (*M* = 5.4 years, *SD* = 0.44) for whom parents and kindergarten teachers completed the SDQ, 77 (81.05%) agreed to participate in the follow-up study at age 14, that is, about 9 years later. Mean age at follow-up was 14.25 years (*SD* = 1.21; 32 females and 45 males). As stated in Brand et al. (2015), participants and non-participants at follow-up did not significantly differ as regards age, gender, sleep profiles or psychological traits (internalizing and externalizing problems, hyperactivity, negative peer-relationship, and prosocial behavior) at baseline. Accordingly, age, gender, sleep profiles, or psychological traits at baseline were not entered as covariates in all statistical equations of the present study.

### Tools Employed at Age Five

Strengths and Difficulties Questionnaire (Goodman, 1997).

Parents and teachers completed the SDQ, which consists of 25 items covering the following five dimensions: internalizing problems, externalizing problems, hyperactivity, peer problems, and prosocial behavior. An overall score can also be derived, with higher scores reflecting more negative psychological functioning. Each scale consists of five items that are rated on a three-point scale ranging from 0 (= not true) to 2 (= certainly true). The sum is calculated to generate subscale scores. In the present study, internal consistency was moderate to high (Cronbach’s alpha = 0.87).

### Tools Participants Employed at Age 14

#### Mental Toughness

Participants were asked to fill in the 18-item Mental Toughness Questionnaire (MTQ18; Clough et al., 2002; German version: Gerber et al., 2012, 2013a,b). The MTQ18 is the short version of the MTQ48 questionnaire (Clough et al., 2002), which has proved to be a valid and reliable instrument in previous research (Gerber et al., 2013b; Perry et al., 2013). Very high correlations exist between the MTQ18 and MTQ48 (Clough et al., 2002; Gerber et al., 2014, 2015a). Answers on the MTQ18 are given on five-point Likert-type scales ranging from 1 (strongly disagree) to 5 (strongly agree). Responses across items are summed, with higher scores reflecting greater MT (Cronbach’s alpha = 0.92).

#### Sleep Disturbances

To assess sleep disturbances, the Insomnia Severity Index (ISI; (Bastien et al., 2001) was employed; this is a 7-item screening measure for insomnia and an outcome measure for use in treatment research. The items, answered on 5-point rating scales (0 = not at all, 4 = very much), refer in part to DSM-IV (Diagnostic and Statistical Manual of Mental Disorders) criteria for insomnia (American Psychiatric Association, 2000) by measuring difficulty in falling asleep, difficulties remaining asleep, early morning awakenings, increased daytime sleepiness, impaired daytime sleepiness, impaired daytime performance, low satisfaction with sleep, and worrying about sleep. Evidence for the validity and reliability of this instrument has been presented previously (Fernandez-Mendoza et al., 2012; Gerber et al., 2016). The higher the overall score, the more the respondent is assumed to suffer from sleep disturbances (Cronbach’s alpha = 0.92).

### Statistical Analysis

First, a series of Pearson’s correlations was performed between SDQ scores (parents’ and teachers’ ratings of children at age five) and participants’ MT and sleep disturbances scores at age 14. Second, two multiple regression analyses (stepwise backward) were performed with MT scores and sleep disturbances (age 14) as dependent variables and SDQ scores (age 5) as predictors. Third, possible gender differences in MT and sleep disturbances at age 14 were calculated with two *t*-tests. Fourth, the bivariate association between MT and sleep disturbances scores at age 14 was examined with a Pearson’s correlation. The nominal level of significance was set at alpha <0.05. Statistics was performed with SPSS^®^ 23.0 (IBM Corporation, Armonk, NY, USA) for Apple Mac^®^.

## Results

Descriptive statistics and bivariate correlations between strengths and difficulties (SDQ) at 5 years and MT and sleep disturbances at 14 years.

All statistical indices are reported in **Table 2** and therefore not repeated in the text again.

**Table 2:** Descriptive statistics and correlations between MT and sleep disturbances at age 14 and teachers’ and parents’ rating of children’s psychological functioning at age 5.

	Dimensions at age 14		Descriptive statistics
		
	Mental toughness	Sleep disturbances	*M* (*SD*)
**Teachers’ ratings**			
Internalizing problems	-0.35^∗∗^	0.24^∗^	1.29 (0.29)
Externalizing problems	-0.36^∗∗^	0.25^∗^	1.39 (0.26)
Negative peer relationship	-0.25^∗^	0.24^∗^	1.25 (0.38)
Hyperactivity	-0.04	0.11	1.56 (0.45)
Prosocial behavior	0.38^∗∗^	-0.21^∗^	1.53 (0.38)
Overall score	-0.19^∗^	0.20^∗^	1.37 (0.21)
**Parents’ ratings**			
Internalizing problems	-0.30^∗∗^	0.21^∗^	1.31 (0.36)
Externalizing problems	-0.24^∗∗^	0.25^∗^	1.36 (0.27)
Negative peer relationship	-0.12	0.10	1.21 (0.29)
Hyperactivity	-0.02	0.03	1.52 (0.41)
Prosocial behavior	0.21^∗^	-0.20^∗^	1.48 (0.39)
Overall score	-0.15	0.12	1.35 (0.20)

Descriptive statistics *M* (*SD*)	22.31 (4.86)	5.64 (3.99)	


*^∗^*p* < 0.05; ^∗∗^*p* < 0.01.*

Lower internalizing and externalizing problems, and higher prosocial behavior, as rated by parents and teachers, were associated with higher MT scores and lower sleep disturbances. Better peer relations and lower overall scores, as rated by teachers, were associated with higher MT scores and lower sleep disturbances. No significant associations were found for hyperactivity (parents’ and teachers’ ratings) or for negative peer relations, as rated by parents.

As regards sleep disturbances, higher sleep disturbances were associated with higher internalizing and externalizing problems, lower prosocial behavior, as rated by parents and teachers, and with more negative peer relationships and higher overall scores, as rated by teachers. No significant associations were found for hyperactivity (parents’ or teachers’ ratings), or for negative peer relationships and overall scores, as rated by parents.

### MT Scores and Sleep Disturbances

The correlation coefficient was *r* = -0.45 (*p* < 0.05); higher MT scores were related to lower sleep disturbances.

### Predicting Mental Toughness and Sleep Disturbances (at 14 Years) from Strengths and Difficulties (SDQ; at 5 Years)

**Table 3** reports the results from the two multiple regression analyses (stepwise backward) with MT scores and sleep disturbances as dependent variables and the strengths and difficulties as predictors (to avoid redundancy and biased calculations, SDQ Total scores were not entered in the equations).

**Table 3:** Overview of the multiple regression analyses (stepwise backward) with MT and sleep disturbances at age 14 as dependent variables and parents’ and teachers’ ratings of the children’s strengths and difficulties (SDQ) at children’s age five as independent variables.

		Non-standardized coefficients		Standardized coefficient					
							
Dimension	Variable	Coefficient beta	Standard error	beta		*p*	*R*	*R^2^*	Durbin-Watson statistics
Mental toughness	Intercept	23.43	1.18	-	19.89	0.000	0.402	0.160	1.67
	Teachers’ internalizing problems	-68.87	33.66	-5.04	-2.05	0.045			
	Parents’ externalizing problems	-61.34	32.80	-5.07	-1.96	0.051			
	Teachers’ externalizing problems	-66.81	33.43	-3.71	-2.00	0.047			
	Teachers’ negative peer-relationship	-65.26	32.50	-3.44	-1.99	0.048			
	Teachers’ Prosocial behavior	64.43	32.50	5.27	1.99	0.049			

	Excluded variables: Teachers’ ratings of hyperactivity; parents’ ratings of internalizing problems, prosocial behavior, hyperactivity, negative peer relationship.								

Sleep disturbances	Intercept	5.98	0.96	-	6.230	0.000	0.339	0.115	1.51
	Parents’ externalizing problems	4.93	2.20	0.320	2.547	0.013			
	Teachers’ externalizing problems	5.08	2.20	0.329	2.284	0.025			
	Teachers’ negative peer relationships	5.956	2.05	0.386	2.902	0.005			

	Excluded variables: Parents’ ratings of internalizing problems, negative peer relationship, prosocial behavior, hyperactivity; teachers’ ratings of internalizing problems, prosocial behavior, hyperactivity.								


Higher Mental toughness scores were associated with lower internalizing problems (parents, teachers), lower externalizing problems, more positive peer relationships, and higher prosocial behavior (teachers). The following variables were excluded from the equation: teachers’ ratings of hyperactivity; parents’ ratings of internalizing problems, prosocial behavior, hyperactivity, and negative peer relationships.

Higher sleep disturbances were associated with higher externalizing problems (parents, teachers), and more negative peer relationships (teachers). The following variables were excluded from the equation: teachers’ ratings of internalizing problems, prosocial behavior, and hyperactivity; parents’ ratings of internalizing problems, negative peer relationships, prosocial behavior, and hyperactivity.

### Gender Differences in MT and Sleep Disturbances

Two *t*-tests (**Table 4**) revealed that females at 14 years, compared to males, had lower MT scores and reported more sleep disturbances.

**Table 4:** Mental toughness scores and sleep disturbances, separated by gender.

	Gender		Statistical analysis
		
	Females	Males	
N	32	45	
Mental toughness	19.33 (3.19)	25.02 (3.51)	*t*(75) = 2.01, *p* = 0.04
Sleep disturbances	7.97 (2.47)	4.41 (2.61)	*t*(75) = 2.21, *p* = 0.03


## Discussion

The key findings of the present study were that lower scores of internalizing and externalizing problems and negative peer-relationships and higher prosocial behavior scores at age five, as rated by parents and teachers, were associated with higher MT scores and lower sleep disturbances scores at age 14. The pattern of results adds to the current literature in an important way in that we were able to shed some light on the origins of adolescent MT by relating this to favorable psychological traits at the age of 5 years.

Three hypotheses were formulated and each of these is considered now in turn.

Our first hypothesis was that positive psychological traits during childhood (lower internalizing and externalizing problems, lower negative peer-relationships, higher pro-social behavior) would be associated with greater MT at 14 years, and this was confirmed. We hold that the present study expands upon previous research in being the first to associate adolescent MT from favorable childhood psychological traits. As shown in **Tables 2** and **3**, both parents’ and teachers’ ratings of children’s lower externalizing, internalizing problems, negative peer relationships and higher prosocial behavior during preschool was associated with higher MT scores during adolescence.

Our second hypothesis was that childhood psychological traits would be associated with lower sleep disturbances scores in adolescence, and this hypothesis also received support. We believe that this pattern of results confirms both the assumed association between positive psychological traits and sleep quality. In this view, there is evidence from longitudinal studies that sleep quality impacts on psychological functioning (Kaneita et al., 2009; Roberts et al., 2009; Hatzinger et al., 2013b, 2014; Roberts and Duong, 2014, 2015; Brand et al., 2015); the results of a meta-analysis indicated that poor sleep predicted symptoms of depression among adolescents, and not vice versa (Lovato and Gradisar, 2014). On the other hand, psychological traits also impacted on sleep patterns (see Brand et al., 2015 for extensive overview), a direction of influence also confirmed in the present study.

Our third hypothesis was that, cross-sectionally, higher MT scores would be related to fewer sleep disturbances, and again data confirmed this. Therefore, the present pattern of results is also consistent with previous findings (Brand et al., 2014a,b, 2016a,b), and underscores the bi-directionality of sleep and psychological functioning.

The data available do not shed any light on why positive psychological traits such as lower internalizing and externalizing problems, lower negative peer-relationships and higher prosocial behavior at 5 years, and as rated by parents and teachers, should be associated with both self-rated increased MT and lower sleep disturbances 9 years later. We know from previous studies (Wong et al., 2010; Fischer et al., 2012; Settles et al., 2012; Shin et al., 2012) that increased psychological issues during childhood also increased the risk of increased psychological issues in adolescence and early adulthood. We also know that personality traits remained fairly stable from childhood to adolescence (Roberts et al., 2001; Moffitt et al., 2011; Shin et al., 2012; Keefer et al., 2013), and in this view, we also know that higher IQ and positive parenting favor the development of resilience in childhood and provide protection under conditions of severe adversity (Masten et al., 1999). In this context, Masten and Tellegen (2012) reported that resilience increased as a function of high quality relationships with parents and other adults (see also Skala and Bruckner, 2014), cognitive quality, social-emotional skills, adaptive resources, planfulness, future motivation, autonomy, adult support, and coping skills (Masten, 2004; Masten et al., 2004), and that resilience often emerged in childhood and endured, but that there were also late bloomers. Sameroff and Rosenblum (2006) emphasized that, in addition to the behavioral and emotional self-regulation characteristic of good mental health and the cognitive self-regulation characteristic of high intelligence, environmental factors such as parenting, peers, and economic conditions may independently and bi-directionally contribute to a child’s resilience.

How should these findings be related to the present study? Our proposal is that lower internalizing and lower externalizing problems, lower negative peer-relationships and higher prosocial behavior might be understood as the behavioral and emotional self-regulation that is characteristic of good mental health, the cognitive self-regulation element as suggested by Sameroff and Rosenblum (2006; Keefer et al., 2013), as well as aspects of the adaptive resources, autonomy and coping skills, as suggested by Masten (2004) and Masten et al. (2004). Additionally, prosocial behavior might be understood as reflecting high quality relationships and stable socio-emotional skills (Masten et al., 2004; Masten and Tellegen, 2012; Skala and Bruckner, 2014). Importantly, in the present study lower internalizing and externalizing problems and higher prosocial behavior were associated with greater MT 9 years later, suggesting therefore considerable stability in level of psychological traits from childhood to mid adolescence (Caspi et al., 2005; Moffitt et al., 2011; Shin et al., 2012).

As regards gender differences, our findings confirmed previous results, in that relative to males, females at age 14 had lower MT scores (Brand et al., 2016a,b) and more pronounced sleep difficulties (Armitage and Hoffmann, 2001; Mong and Cusmano, 2016).

Despite the novelty of the findings, several limitations warrant against their overgeneralization. First, the sample size was small, and a larger sample would have provided greater statistical power and may therefore have revealed other significant associations. Second, the pattern of results might have emerged due to further latent, but unassessed dimensions, which might have biased two or more variables in the same or opposite directions. This holds particularly true, as it is conceivable that latent MT traits and sleep patterns at the age of five might have conferred to the MT and sleep disturbances scores at the age of 14. Further, for instance, parenting style was not assessed at both time points. In this regards, there is evidence that children’s and also adolescents’ behavior and sleep are not independent of family functioning, parenting style (Brand et al., 2009), or parents’ sleep patterns (Brand et al., 2009; Kalak et al., 2012; Bajoghli et al., 2013). Third, no neurophysiological data were gathered at the second time point; previous studies have shown that, for instance, cortisol secretion remained stable over time (Hatzinger et al., 2013a), while cortisol secretion is not related to sleep patterns (sleep-EEG; actigraphy) 12 months later, suggesting therefore that cortisol secretion may also vary as a function of current physical and psychological processes. Fourth, sleep at age 14 was only assessed subjectively. Fifth, only the MT overall score was applied (MTQ18); employing the long version (MTQ48) would have allowed a more fine-grained analysis of the associations between childhood and adolescent psychological functioning. In this regard, we underscore that the current interpretations rely on the assumption that psychometric properties of the MTQ18 used with adolescents are acceptable. Sixth, it is conceivable that the strengths and difficulties at the age of 5 years are the result of psychosocial development from very first infanthood to preschool-age, suggesting therefore that the origins of adolescents’ MT might be routed even earlier in development. Seventh and last, given that the concept of MT has gained increasing attention for its interest utility among elite and non-elite athletes to explaining a broad range of coping, motivation, self-esteem and health-related behavior (see Introduction and **Table 1**), future research might focus on the causal relation between MT traits and such constructs as reported in **Table 1**.

## Conclusion

Positive psychological traits as reflected in lower internalizing and externalizing problems and higher prosocial behavior at age five was associated with higher MT scores and lower sleep disturbances scores at age 14. The pattern of results suggests that positive psychological traits in childhood seemed to lay the foundation for adolescent MT. This is important because MT has proved to be a stress resilience factor during both adolescence and young adulthood.

## Author Contributions

Study design: DS, MH, MG, SL, PC, SP, KK, AW, EH, and SB; data gathering: DS, MH, SP, AW, and SB; data analysis: DS, MG, SL, and SB; interpretation of the data: DS, MH, MG, SL, PC, SP, KK, AW, EH, and SB; writing the first draft: DS, MG, EH, and SB; integration of authors’ comments: DS, MG, SL, and SB; final manuscript: DS, MH, MG, SL, PC, SP, KK, AW, EH, and SB.

## Conflict of Interest Statement

The authors declare that the research was conducted in the absence of any commercial or financial relationships that could be construed as a potential conflict of interest.

The handling Editor declared a past co-authorship with one of the authors SB and states that the process nevertheless met the standards of a fair and objective review.
